# A study on the relationship between the intensity of mobile social media use and the quality of friendships among Chinese youth in the context of social media fatigue

**DOI:** 10.3389/fpsyg.2026.1806533

**Published:** 2026-06-29

**Authors:** Hao Sun, Mingche Li

**Affiliations:** 1 School of International Politics and Communication, Beijing Language and Culture University, Beijing, China; 2 School of Journalism and Communication, Qufu Normal University, Qufu, China

**Keywords:** Chinese youth, friendship quality, friendships and relationships, mobile social media, social media fatigue

## Abstract

**Introduction:**

Healthy friendships are crucial for the psychological development of young adults. Today, mobile social media has become a vital tool for young people to establish and maintain friendships, offering significant convenience for developing friendships. However, as usage intensity increases, the resulting social pressure and negative emotions have gradually become issues regarding young people’s interactions with friends that cannot be ignored.

**Methods:**

This study employs a mixed-methods research approach combining quantitative and qualitative methods to explore new changes in friendship quality resulting from online socializing under social media fatigue among Chinese young people, along with the underlying causes of these changes.

**Results:**

Findings reveal that social media fatigue induces changes in friendship maintenance behaviors among Chinese young people, primarily manifested as reduced willingness to interact, diminished responsiveness, and a tendency toward deeper engagement.

**Discussion:**

During behavioral adjustment, Chinese youth progressively filter out superficial weak ties, redirecting greater energy toward a limited number of intimate relationships.

## 1 Research background

In 2017, the Central Committee of the Communist Party of China and the State Council issued the *Medium and Long-Term Youth Development Plan (2016–2025)*, emphasizing the importance of young people’s mental health. Friendship is a vital social relationship for young people, significantly influencing their psychological development. High-quality friendships can strongly predict lower levels of depression among young people (Li Rong et al., 2023). Historically, young people primarily relied on offline interactions to maintain friendships. However, in recent years, with the advancement of internet technology, mobile social media has gained increasing popularity, becoming a new avenue for young people to establish, maintain, and foster friendships. Data from the Pew Research Center indicates that nearly two thirds of young people have made new friends through social media, with one third engaging in daily online interactions with these friends (Fu Senhui, 2020). This proactive use of social networking sites can effectively enhance the quality of friendships among young people (Li Xiaoran, 2024).

However, as mobile social media increasingly permeates daily life it fosters a state of being “permanently online, permanently connected,” subjecting users to persistent emotional, psychological, and energetic strain alongside the convenience of instant communication (Rong Rong and Zhao Wenjing, 2024). People’s enthusiasm for social media is waning, and the number of active users is gradually declining (Zhao Youlin et al., 2025). This phenomenon of diminishing interest in, or reduced use of, social media is termed “social media fatigue” by academics. Against this backdrop, examining whether youth friendships are being newly influenced by social media holds significant implications. Such research can guide platforms in better directing young people toward appropriate online usage and preventing fatigue, while also helping them to navigate interpersonal relationships effectively and reduce psychological issues.

## 2 Research questions

### 2.1 Online socializing enhances friendship quality

Friendship is a voluntary interpersonal relationship formed through individual choice and plays a crucial role in personal growth and socialization. Research on friendship can be divided into three levels: The first level concerns the existence of friendship, that is, whether there is a voluntary interpersonal relationship between individuals; the second level concerns the scope of friendship, that is, the number of friends with whom there is mutual recognition, which this paper refers to as the breadth of friendship; the third level concerns the quality of friendship, that is, the degree to which friends support and accompany one another, which this paper refers to as the depth of friendship (Zou Hong et al., 1998). Research indicates that the quality of friendship significantly negatively predicts loneliness and depression while alleviating social anxiety (Tian Lumei et al., 2012; Levine et al., 2015), and significantly positively predicts extraversion, prosociality, conscientiousness, and emotional stability (Yang Lizhu et al., 2012). Consequently, friendship quality is often regarded as a key indicator for assessing an individual’s mental health status and level of social adaptation.

With the rise of social media platforms like QQ and WeChat, friendship interactions have expanded from offline to online spaces, making the factors influencing friendship quality more complex and diverse. Most scholars agree that the usage of social networking sites shows a significant positive correlation with friendship quality and a significant negative correlation with loneliness (Zhou Kuizong et al., 2017). On the one hand, mobile social media provides a more convenient new platform for emotional communication between individuals, facilitating self-presentation behaviors (Wang Wei et al., 2017) that directly enhance offline friendship quality (Zhang Xiaozhou et al., 2020). On the other, both positive and authentic self-presentation can help individuals gain more positive online feedback (Cui Xixi et al., 2016) and social support (Wang Wei et al., 2017), thereby indirectly enhancing friendship quality offline.

Existing research has effectively illuminated the promotional effects of online socializing on friendship quality and its underlying mechanisms. However, in recent years, the new lifestyle of “permanent online presence and constant connectivity” enabled by mobile social media, while enriching users’ emotional experiences, has increasingly become an invisible source of pressure and constraint (Rong Rong and Zhao Wenjing, 2024). This negative emotional state may prompt users to disengage from online communities and reduce online interactions with friends (Huang Honghui, 2020). In the context of social media fatigue, the question of how online socializing will affect individual friendship quality remains worthy of consideration.

### 2.2 Social media fatigue suppresses online socializing

Social media fatigue leads users to actively reduce the frequency of their social media usage, diminish their willingness to interact, and even withdraw from social media platforms (Guo Jia and Cao Fenfang, 2018). Existing research refers to this phenomenon as social media “nonuse” (Huang Ying, 2018).

Specifically, social media “nonuse” encompasses negative behaviors such as ignoring messages, lurking (silent browsing), blocking (content filtering), resisting (complaining or dismissing), or even quitting (uninstalling or deactivating features) (Liu Luchuan et al., 2017). Current academic research on social media “nonuse” behavior primarily adopts the following perspectives: First, using the stress–source–outcome (SSO) framework, studies reveal that social media fatigue mediates the relationship between social overload, information overload, and intentions to discontinue social media use (Niu Jing and Chang Mingzhi, 2018; Maier C et al., 2012); second, based on stress theory, it points out that role stress and relational stress encroach on private space, leading to user fatigue and a return to offline paths (Xue Jing and Hong Jiewen, 2020; Zhao Qinan, 2019); third, the stimulus–organism–response (SOR) paradigm suggests that environmental stimuli trigger internal psychological responses—such as emotions, attitudes, and cognition—which subsequently drive users toward a state of nonuse (Dai Bao et al., 2020; Luqman et al., 2017). Thus, social media nonuse emerges as a direct consequence of social media fatigue, effectively inhibiting individuals’ online social engagement.

Existing research indicates that social media fatigue may prompt users to “disengage” from social media platforms, yet it has paid little attention to the actual changes this “nonuse” behavior brings to individuals’ lives. Therefore, this study employs the SOR theory to explore new patterns in the relationship between the intensity of mobile social media usage and friendship quality among young Chinese in a state of fatigue. It aims to provide this population with theoretical foundations with which to use social media appropriately and maintain healthy friendships while also offering practical insights for platform optimization and user fatigue prevention. Additionally, it seeks to contribute relevant data for other similar research in countries and regions.

## 3 Theoretical model and research hypotheses

### 3.1 The stimulus–organism–response (SOR) theoretical model

**Figure 1:**
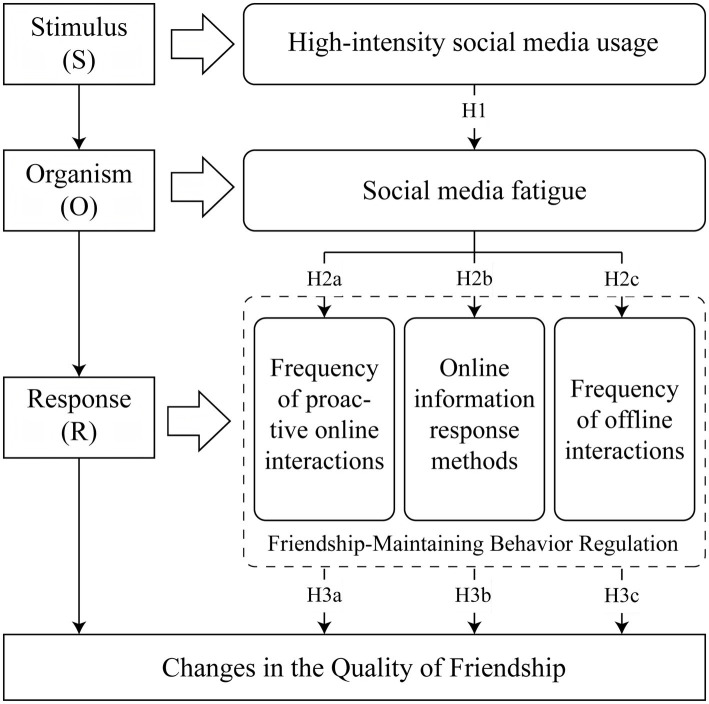
A model of the relationship between mobile social media usage intensity, social media burnout, and friendship quality among young people based on SOR theory.

The SOR theory posits that environmental stimuli induce changes in an organism’s internal cognition and emotions, thereby influencing its external behavioral manifestations (Pan Taotao and Lü Yingjie, 2022). For some users, negative effects such as information overload and social overload experienced while using mobile social media can lead to feelings of stress and burnout (Liu Luchuan et al., 2018), while simultaneously heightening their expectations for friendships. Against this backdrop, users may consciously avoid social media and proactively adjust how they interact with friends (Zhao Qinan, 2019), leading to corresponding changes in friendship quality. The specific pathway is illustrated in Figure 1. Furthermore, existing research has validated the model’s effectiveness in explaining changes in social media users’ psychological states and behavioral responses (Gan Chunmei and Ming Xinyu, 2023). Therefore, this study aims to use the SOR theoretical framework to elucidate how burnout introduces new dynamics in the relationship between social media usage intensity and friendship quality among Chinese young people, as well as the underlying reasons for these changes.

### 3.2 Research hypotheses

With the expansion of information resources, users increasingly rely on intensive social media use to access more information than ever before to meet their needs. However, excessive use not only leads to information overload but also causes issues such as redundant social relationships and privacy breaches, imposing dual pressures on users’ physical and mental well-being (Liu Luchuan et al., 2017). Based on this, the following hypothesis is proposed:

*H1*: The intensity of mobile social media usage positively influences social media fatigue.

“Online silence” refers to passive social behaviors such as delayed responses or reading messages without replying that occur when users engage with social media (Rong Rong and Zhao Wenjing, 2024). The culture of “permanent online presence and constant connectivity” on social media imposes heavy burdens such as social fatigue on users, giving rise to online silence and even digital disconnection (Chen Xuewei and Zhang Pengxia, 2021). When users’ social needs cannot be met online, they become more inclined to seek support from friends and family in their immediate surroundings (Zhao Qinan, 2019). Based on this, Hypothesis 2 is proposed:

*H2a*: Social media fatigue negatively affects the frequency of proactive online interactions.

*H2b*: Social media fatigue positively influences negative online message responses.

*H2c*: Social media fatigue positively influences offline interaction frequency.

Proactive communication fosters mutual understanding among friends, thereby strengthening the bonds of friendship (Lian Shuailei et al., 2017). Conversely, when users resort to online silence in interpersonal relationships, both parties experience psychological pressure and tend to avoid interaction to some extent (Rong Rong and Zhao Wenjing, 2024). Furthermore, existing research confirms that face-to-face relationships provide greater emotional support than virtual ones (Chen Fuping et al., 2018). Based on this, the third hypothesis is proposed:

*H3a*: Frequency of proactive interaction online positively influences friendship quality.

*H3b*: Negative online communication responses negatively affect friendship quality.

*H3c*: Frequency of offline interactions positively influences friendship quality.

## 4 Research design

### 4.1 Research participants and methods

The selection of research subjects primarily considered the definition of youth. Different organizations and institutions define youth age ranges differently. The *Medium and Long-Term Youth Development Plan (2016–2025)* issued and implemented by the Central Committee of the Communist Party of China and the State Council in 2017 specifies youth as ranging from 14 to 35 years old. Individuals under 18 were excluded from this study because their usage of mobile social media may be constrained by academic pressures or parental supervision, making it difficult to align with their autonomous preferences. Therefore, the study focuses on Chinese young people aged 18–35 who experience social media fatigue. This study employs a mixed-methods approach combining quantitative and qualitative research to explore how online social interactions among young adults in social media fatigue contexts may bring about new changes in friendship quality.

### 4.2 Quantitative research based on questionnaire surveys

#### 4.2.1 Data collection

In this study, the questionnaire survey aims to test the aforementioned hypotheses and provide a fundamental basis for designing the interview outline. The questionnaire was developed by the researchers and consists of five modules: personal background information (as shown in Table 1); mobile social media usage patterns; experiences of social media fatigue; friendship maintenance behavioral regulation; and perceptions of friendship quality (see Table 2 for a list of example questions from each module). The questionnaire primarily featured single-choice and multiple-choice questions, supplemented by a small number of open-ended items. Prior to formal release, the author conducted preliminary back testing and made appropriate adjustments to the questionnaire content based on valid feedback to ensure reliability and accuracy. The questionnaire was distributed entirely online, yielding 533 responses. After sorting and excluding invalid responses, a total of 511 valid questionnaires were obtained.

**Table 1:** Basic sample information.

Basic information	Item	Number	Percentage
Gender	Male	239	46.77%
	Female	272	53.23%
Age	18–25 years old	388	75.93%
	26–35	123	24.07%
Education level	High school or below	6	1.17%
	College	75	14.68%
	Bachelor’s Degree	334	65.36%
	Master’s and above	96	18.79%
Occupation	Student	366	71.62%
	Civil Servant/Public Institution Employee	32	6.26%
	Corporate Employees	77	15.07%
	Freelancers	29	5.68%
	Other	7	1.37%
Income level	Below ¥2,000	193	37.77%
	¥2,000–¥4,000	190	37.18%
	¥4,001–¥6,000	69	13.5%
	¥6,001–¥8,000	36	7.05%
	Over 8,000 yuan	23	4.5%

**Table 2:** Sample questionnaire questions.

Dimension	Question
Mobile social media usage	How much time do you spend on social media platforms each day?
Experiencing social media fatigue	Have you ever felt exhausted, frustrated, or like you just wanted to escape from social media?
Regulation of friendship-maintaining behaviors	How often do you reach out to your friends online?
	How often do you engage in passive-aggressive behavior (such as reading a message but not replying, delaying a reply, or giving a perfunctory response)?
	How much do you look forward to receiving messages from your friends?
	How often do you meet up with your friends in person?
Perception of the quality of friendship	Does using mobile social media when you are feeling burnt out affect the quality of your friendships?
	Overall, how would you describe your relationship with your friends right now?

#### 4.2.2 Reliability and validity testing

The questionnaire for this study was designed primarily around several variables, including mobile social media use, social media burnout, friendship maintenance behaviors, and perceived friendship quality, with a focus on examining the relationships among these variables. Since the questionnaire includes scale-type items only for certain questions in the modules on friendship maintenance behavior moderation and perceived friendship quality, while the remaining variables are measured using single non-scale items—and thus do not constitute a strict scale in the traditional sense—Cronbach’s *α* and KMO coefficients are used solely as reference indicators for data analysis, rather than as rigorous validation of the questionnaire’s internal reliability or validity. A reliability and validity analysis was conducted on the relevant scale items, yielding Cronbach’s *α* = 0.625 and KMO = 0.654. Additionally, Bartlett’s sphericity test reached statistical significance (*p* < 0.001), as shown in Table 3. Consequently, the questionnaire data provide a sufficient foundation for subsequent correlation analysis.

**Table 3:** Reliability and validity tests.

Cronbach’s alpha coefficient	KMO coefficient	*p*-value
0.625	0.654	0.000

#### 4.2.3 Hypothesis testing

The questionnaire data are imported into SPSS and Pearson correlation analyses are conducted for the two variables specified by each hypothesis: If *p* < 0.05, this indicates a significant correlation between the two variables. Under this condition, a positive correlation coefficient signifies a positive relationship between the variables, while a negative coefficient indicates the opposite. The specific analysis results are shown in Table 4. Except for H2c, which was not supported, all other hypotheses passed the test.

**Table 4:** Results of Pearson correlation analysis.

Item	Hypothesis	Correlation coefficient	*p*-value	Correlation relationship	Test result
H1	Usage intensity → Social media fatigue	0.234	0.000	Positive correlation	Supported
H2a	Social media fatigue → Frequency of online proactive interactions	−0.322	0.000	Negative correlation	Supported
H2b	Social media fatigue → Negative online responses	0.424	0.000	Positive correlation	Supported
H2c	Social media fatigue → Frequency of offline interactions	−0.113	0.010	Negative correlation	Not supported
H3a	Frequency of active online interaction→ Friendship quality	0.159	0.000	Positive correlation	Supported
H3b	Negative online responses → Friendship quality	−0.093	0.037	Negative correlation	Supported
H3c	Offline interaction frequency → Friendship quality	0.377	0.000	Positive correlation	Supported

### 4.3 Qualitative research based on in-depth interviews

To further explore the mechanisms and outcomes of the impact of burnout on friendship quality among Chinese young people, this study conducted semi-structured in-depth interviews with 25 participants. The research delves into the causes of social media burnout within this demographic and its moderating effects on friendship maintenance behaviors, examining how friendship quality evolves under burnout influences. A three-level coding framework was established to construct the relevant model. It has been confirmed that none of the 25 respondents participated in the questionnaire survey; therefore, the interview data is authentic and valid. The interview outline was developed based on relevant literature and adjusted during the process, focusing on the following four aspects (see Table 5 for a list of example questions from each dimension): First, the current status of mobile social media usage, including commonly used platforms, frequency of use, and usage patterns; second, social media fatigue, encompassing triggers of fatigue, psychological experiences, and coping strategies; third, changes in friendship maintenance behaviors, such as communication expectations, response patterns, and interaction frequency; and fourth, perceptions of friendship quality, specifically whether and how friendship quality changed after experiencing fatigue.

**Table 5:** Interview outline.

Dimension	Question
Mobile social media usage	Which social media platforms do you mainly use? How often do you use them?
	What do you usually do on these platforms? Do you often interact with friends online?
Experiencing social media fatigue	Have you ever felt exhausted, frustrated, or like you just wanted to escape from social media? Can you share a specific experience?
	When you feel burned out, how do you adjust or cope with that feeling? Do you cut back on your social media use?
Regulation of friendship-maintaining behaviors	Does burnout affect how often you connect with friends online? Or do you find yourself being more selective about your existing relationships?
	Has burnout changed your expectations of your friends or the way you respond to them? For example?
	Has your in-person interaction with friends been affected by social media fatigue? Could you elaborate?
Perception of the quality of friendship	How do you usually measure the quality of your relationships with friends?
	Do you feel that the quality of your friendships has been affected by social media fatigue? Is this effect positive or negative? What led you to this conclusion?

The 25 participants hailed from multiple provinces and municipalities across China, including Shandong, Anhui, Yunnan, and Hubei. With participants’ consent, the entire interview process was audio-recorded, subsequently transcribed and organized to yield detailed and valid interview data. For organizational purposes, participants were coded by gender as M1, M2, W1, W2, with their basic information summarized in Table 6.

**Table 6:** Basic information of respondents.

No.	Code	Gender	Age	Education	Occupation	Income level (¥)
1	M1	Male	21	Associate Degree	Student	2000
2	M2	Male	21	Undergraduate	Student	2,500–3,000
3	M3	Male	26	Bachelor’s Degree	Corporate employee	5,500
4	M4	Male	22	Undergraduate	Student	1800
5	M5	Male	30	Bachelor’s Degree	Financial accounting	3,200
6	M6	Male	35	Bachelor’s Degree	Telecommunications industry	8,000
7	W1	Female	23	Undergraduate	Student	2000
8	W2	Female	21	Undergraduate	Student	2000–3,000
9	W3	Female	19	Undergraduate	Student	2,300
10	W4	Female	18	High School	Student	No steady income
11	W5	Female	27	Master’s Degree	Unemployed	3,000
12	W6	Female	22	Undergraduate	Student	1,500
13	W7	Female	20	Undergraduate	Student	2,500
14	W8	Female	27	PhD	Student	2000–3,000
15	W9	Female	26	Master’s Degree	Student	2000
16	W10	Female	23	Master’s Degree	Student	1,500
17	W11	Female	20	Undergraduate	Student	3,000
18	W12	Female	23	Undergraduate	Student	2000
19	W13	Female	23	Undergraduate	Student	2000
20	W14	Female	24	Technical College	Freelancer	1,600
21	W15	Female	22	Undergraduate	Unemployed	4,000
22	W16	Female	20	Undergraduate	Student	2000
23	W17	Female	30	Bachelor’s Degree	Government agency contract worker	4,600
24	W18	Female	30	PhD	International student	8,000–9,000
25	W19	Female	31	Bachelor’s Degree	Engineer	5,000

#### 4.3.1 Open coding

Through sentence-by-sentence annotation and screening of the original interview data, this study extracted 34 initial concepts. Analysis and clustering of these initial concepts yielded 11 categories, including reduced willingness, negative responses, and decreased interaction. The initial concepts and their corresponding categories are shown in Table 7.

**Table 7:** Open coding results.

Category	Initial concept	Representative original statements
High-intensity usage	Long duration of use	I use it whenever I’m awake.
	Multiple usage methods	I use these platforms for chatting, posting short videos, and looking up life hacks.
Internal factors	Negative emotions	When people I care about do not like my posts, it eats away at me.
	Low sense of worth	I feel like I’ve wasted a lot of time without gaining anything in return.
	Communication barriers	Through online chats, I cannot tell how the other person is feeling.
	Privacy concerns	I will not post photos that clearly reveal where I’m hanging out.
	Distractions	After spending too much time scrolling through Douyin, I find it hard to concentrate on studying.
External factors	Social overload	Some people I’ve added as friends are essentially strangers to me.
	Social pressure	If you do not reply to someone, you feel pressured.
	Platform limitations	The content pushed is largely repetitive, offering nothing truly fresh or eye-opening.
	Work pressure	At work, the company’s pace is fast, and I need to respond to a large volume of messages.
	Information overload	During this period, I’m constantly bombarded with an overwhelming amount of information.
Declining engagement	Reduced desire for messages	When I’m feeling burnt out, I wish no one would message me—not even good friends or bad friends, those who make me feel comfortable or uncomfortable.
Cautious responses	Focus on wording	I now strive to make my words unambiguous and avoid breaking them into segments. I also use emojis as much as possible, just to prevent others from taking things out of context.
Negative responses	Half-hearted replies	When someone sends me a long message, I might only reply with a few sentences.
	Delayed responses	I’ll pretend not to see it and reply the next day.
	Read but not replied	Sometimes I feel a bit awkward, so I basically just read and do not reply.
	Reducing output	Subjectively, my output might decrease, or even stop entirely.
Reducing social interaction	Less interaction	Sometimes I might go days without sending a single message.
	Digital disconnection	When I first started middle school, I deleted my WeChat account, and I also deleted my QQ.
	Account deactivation	What I’ve done is deactivate my Xiaohongshu account and then registered a new one.
	Focus on reality	Go out for a walk and shift your focus.
	Switch content	I might explore more fulfilling content areas.
	Switch platforms	Online, I’ll shift from WeChat to Xiaohongshu—places where I do not need to interact with acquaintances.
Prioritize depth	Shift toward strong relationships	I’ll be more proactive in responding to messages from friends. I feel that the friends I’ve kept after filtering are the ones I value most.
	Offline or voice calls	For friends who live nearby, we might switch to meeting in person.
Filtering relationships	Creating a secondary account	For example, when I first started college, I created a secondary WeChat account.
	Set permissions	Nowadays, I mostly share Moments updates with specific groups.
	Selective connections	I feel that the relationships and friends I maintain now are likely those with more overlap in work and life, and thus more meaningful connections.
Quality of strong relationships	Strengthening strong bonds	By eliminating unnecessary social time and investing more effort into nurturing relationships you believe can deepen, your connections will undoubtedly improve.
	Weakening strong bonds	One or two friends who were particularly close to me before I quit my job have since found new friends.
	Strong relationships remain unchanged	I feel that relationships already characterized by high quality will not be significantly affected.
Weak relationship quality	Weak ties fade	At most, I might reduce social interactions with people I wasn’t very close to begin with.
	Weak relationships remain unchanged	Relationships with less familiar people might just stay where they are.

#### 4.3.2 Main axis coding

Based on the 11 specific categories obtained through open coding, this study logically analyzed and grouped them into four main categories: social media usage; social media fatigue; friendship maintenance behaviors; and perceived friendship quality. The main categories, corresponding specific categories, and initial abstractions are shown in Table 8.

**Table 8:** Main axis coding results.

Primary category	Specific categories	Initial concept
Social media use	High-intensity use	Extended duration and diverse methods of usage
Social media fatigue	Internal Factors	Negative emotions, low sense of value, communication barriers, privacy concerns, attention distraction
	External factors	Social overload, social pressure, platform flaws, real-world stress, information overload
Friendship maintenance behavior	Reduced willingness	Reduced information expectations
	Cautious responses	Focus on expression methods
	Negative responses	Superficial replies, delayed responses, read receipts without replies, reduced output
	Reduced interaction	Reduced interaction, digital disconnection, account deactivation, focusing on real life, switching content, switching platforms
	Prioritize depth	Shift toward strong relationships, offline or voice communication
	Filtering relationships	Create secondary accounts, set permissions, selectively connect
Friendship quality	Strong relationship quality	Strengthen strong relationships, maintain strong relationships
	Weak relationship quality	Weak relationship fading, weak relationship unchanged

#### 4.3.3 Selective coding

**Figure 2:**
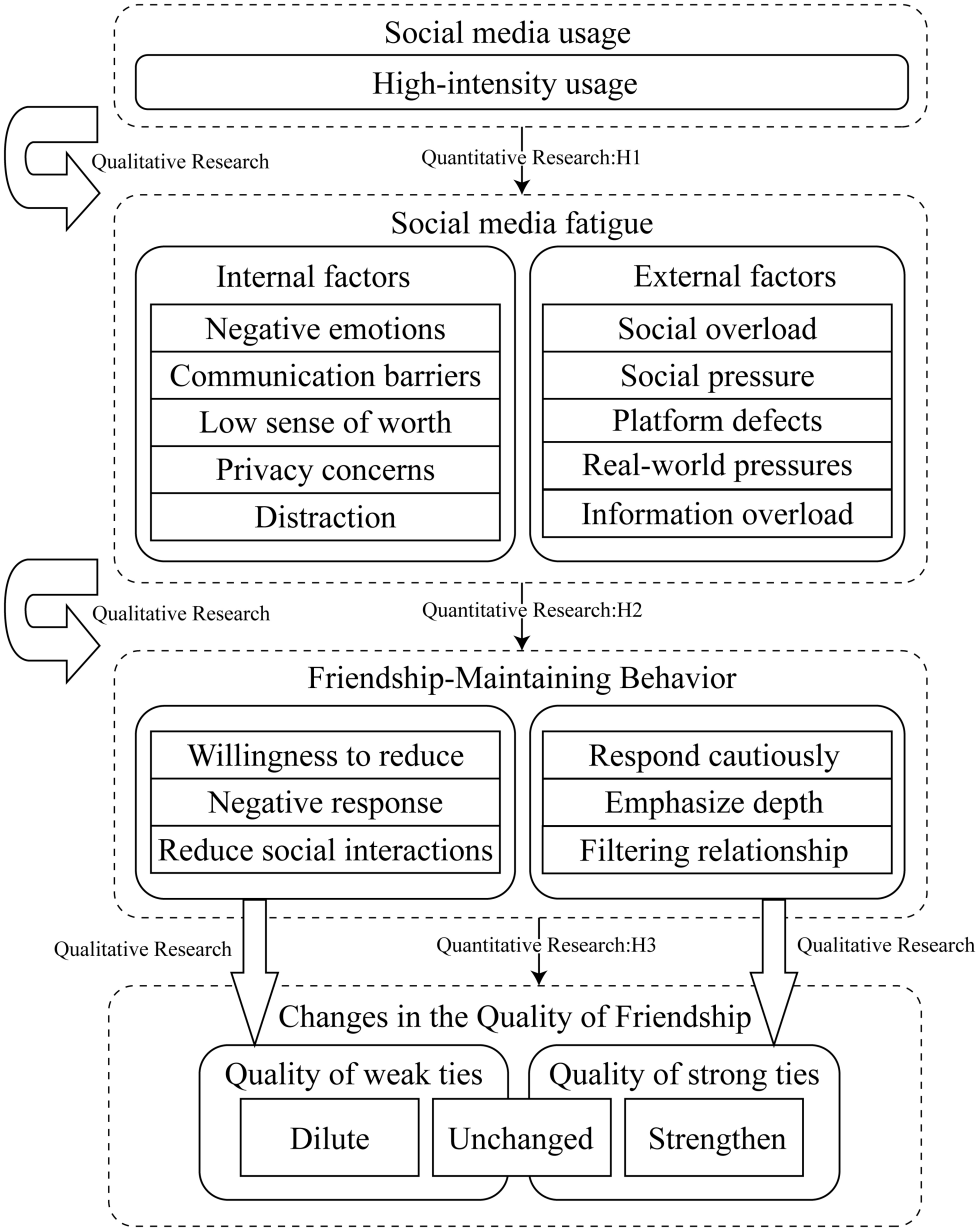
A model of how social media fatigue affects the quality of friendships among Chinese young people.

Through in-depth analysis and selective coding of 46 initial concepts, 11 specific categories, and 4 main categories, the core research category was identified as “How social media fatigue affects friendship quality by altering friendship maintenance behaviors.” Centered on this core category and integrating quantitative research findings, a model illustrating how social media fatigue impacts friendship quality among the youth of China was constructed, as shown in Figure 2.

## 5 Research findings

Based on the questionnaire data and interview materials from 25 respondents, it is evident that the experience of Chinese young people shifts in their perception of friendship quality when suffering social media fatigue. The key to this shift lies in the specific behavioral adjustments made to maintain friendships as a result of this fatigue. After experiencing fatigue, Chinese young people exhibit a reduced willingness for online socializing, tending to manage friendships through passive or cautious responses and decreased interaction while placing greater emphasis on relationship depth. Against this backdrop, they progressively delineate boundaries between “close relationships” and “superficial relationships,” deepening strong bonds while allowing weaker ties to fade.

### 5.1 Fatigue-induced adjustments in friendship maintenance behaviors

In the course of online socializing, China’s youth population has developed a state of social media fatigue due to their intensive social media use, leading to issues such as information overload, social overload, and privacy concerns. This fatigue has, to some extent, influenced the way young Chinese interact with friends online and even extends to offline interactions. Specifically, this fatigue diminishes individuals’ willingness to engage with friends and fosters negative attitudes during interactions. However, it also prompts them to adjust their social behaviors, shifting toward more cautious and deeply rooted friendship maintenance patterns.

#### 5.1.1 Reduced willingness to interact and respond

**Figure 3:**
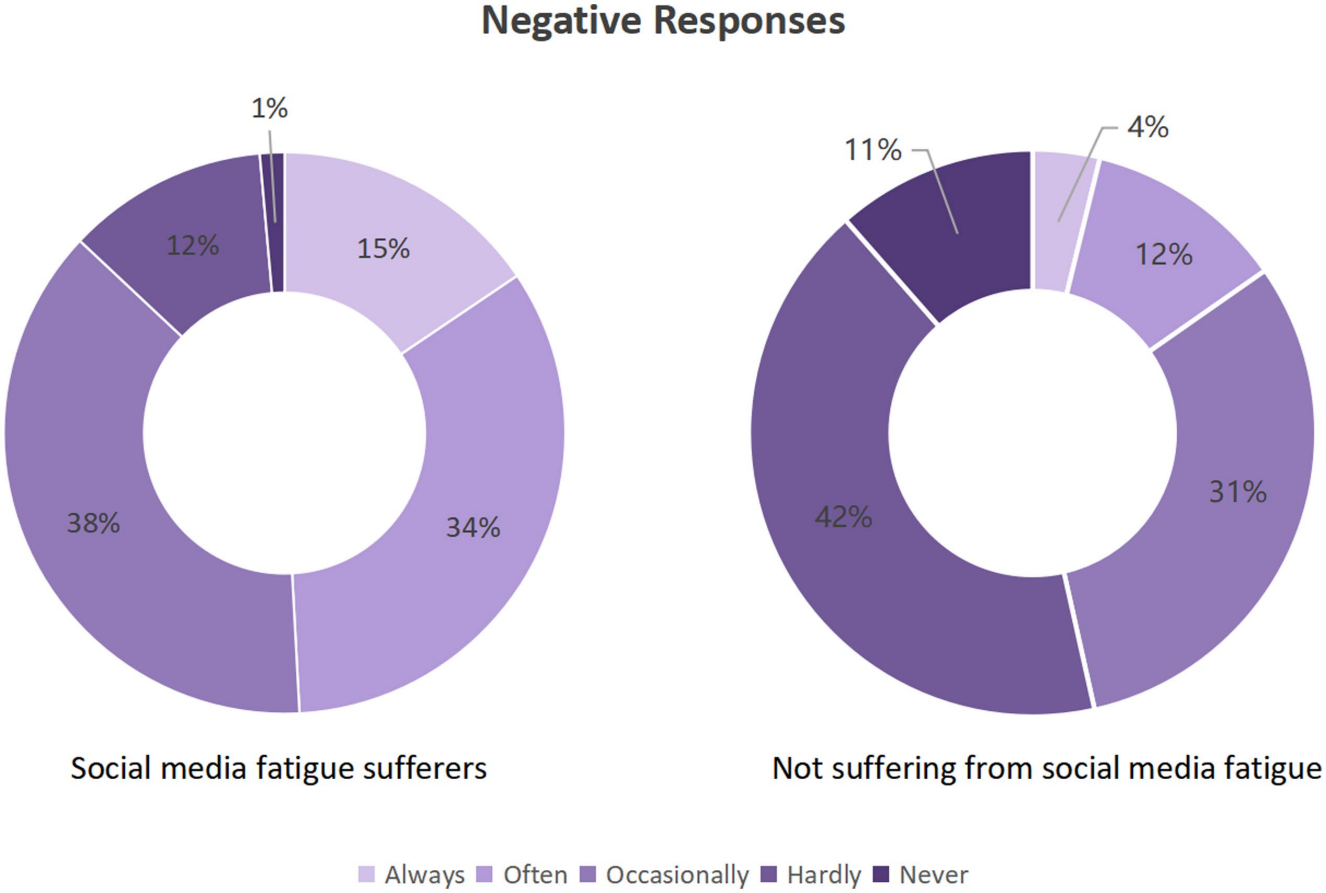
The probability that burnout and non-burnout individuals “always” or “often” engage in passive-aggressive behavior.

In social media fatigue scenarios, individuals show a decreased willingness to invest in relationship maintenance and adopt a passive mindset toward social interactions. A significant portion of Chinese young people noticeably reduce their attention and anticipation toward friends’ messages when feeling fatigued. The survey results indicate that social media burnout is positively correlated with negative online behavior; young people experiencing social media burnout are 33% more likely to engage in negative online behavior “always” or “often” than those who do not experience burnout, as shown in Figure 3. On the one hand, regarding private conversations, they no longer check messages as frequently or respond instantly as before; some even activate their phones’ Do Not Disturb mode to avoid the pressure of replying. On the other, regarding public-space “etiquette,” many young Chinese avoid redundant interactions by closing their Moments feeds, deactivating their accounts, or uninstalling apps. Of the 25 respondents, 16 mentioned that even when they see messages from friends while feeling exhausted, they might respond half-heartedly or delay replies due to a lack of energy or boredom, or even resort to “read but not replied” tactics. This approach is not born out of disregard for friendships but rather stems from depleted psychological energy and a temporary rejection of social demands.

Some people constantly put themselves out there in social interactions, but since I’m already replying to messages under work pressure, I have no energy left for friends like that. I’ll pretend not to see it and reply the next day with something like, “Sorry, I fell asleep yesterday. Do you still need me to comfort you?” (W8).

The weakening of interaction willingness and responsiveness affects friendships of varying strengths differently. For weak ties, interactions often depend on frequent messaging and immediate responses. Thus, a detached attitude during burnout more readily leads to broken contact and fading bonds. In strong relationships, nearly half of the respondents indicated that while burnout-induced behavioral shifts may cause short-term misunderstandings or distance, mutual understanding and tolerance between partners limit the substantive impact of such withdrawal.

I’d also tell everyone during my low points how exhausting real life is, but since they did not cut off the spark or delete me as a friend—and even when they knew I’d read messages without replying, they still messaged me to keep the spark alive—I feel we still have an online bond (M3).

#### 5.1.2 Shifting toward deeper connections

Social media fatigue does not simply lead to complete social withdrawal; it triggers a shift in friendship maintenance patterns. Specifically, young Chinese individuals in fatigued states exhibit distancing and filtering of superficial relationships while increasingly directing limited energy toward deepening connections with a select few close friends. This selective investment reflects a shift in friendship networks from breadth to depth, demonstrating how fatigue reshapes relational patterns. The quantitative study results show that 79 of the 354 participants experiencing burnout believed that burnout prompted them to use more in-depth forms of communication—such as voice calls and face-to-face interactions—in place of text-based communication, thereby improving the quality of their friendships. In the qualitative study, half of the respondents also indicated that while they actively reduced their responses to casual acquaintances during periods of burnout, they still maintained or even strengthened communication with close friends.

There are many things I do not want to share with people around me—like roommates or classmates—but I can express them through text on WeChat with this friend. She gives me positive, encouraging responses. Chatting with her makes me feel happy and joyful; There’s this positive cycle that helps alleviate social fatigue (W9).

This shift in interaction patterns has dual effects. On the one hand, it weakens the maintenance of weak ties, leading to a certain contraction in the social networks of China’s youth. On the other, it helps strengthen the stability and cohesion of strong ties, thereby enhancing the quality of core friendships to some extent. In other words, fatigue drives a process of “refining friendships” among Chinese young people. This phenomenon reveals that fatigue is not a singular negative variable but may play a dual role in both destruction and construction.

### 5.2 Behavioral changes promote differentiation in friendship quality

Quantitative research has found that more than half of young people in China believe social media fatigue has had either a positive or negative impact on the quality of their friendships. Qualitative research, however, further indicates that this impact does not apply uniformly across the entire network of friendships. Building upon the behavioral adjustments driven by friendship fatigue to maintain relationships, friendship networks among young Chinese exhibit a trend toward further differentiation. Specifically, behavioral adjustments resulting from burnout tend to drive a divergence in the quality of strong and weak social ties to some extent: Strong relationships tend toward stability or even deepening after undergoing selection and intensified interaction, while weak relationships gradually stagnate or fade due to a lack of interaction momentum. Thus, the altered interaction patterns resulting from friendship fatigue further influence the quality of relationships within the friendship networks of young Chinese, leading to a “stable core-shrinking periphery” pattern.

#### 5.2.1 Stabilization and deepening of strong relationships

Research findings indicate that as online socializing becomes more generalized, individuals increasingly focus their energy on a small number of truly significant friends. Although burnout has led to a general decline in the frequency of online social interactions among Chinese young people, and even an attitude of indifference, the diversity of maintenance strategies for strong friendships and the stability of the emotional foundation between partners result in relatively high tolerance for each other’s behavioral shortcomings. Simultaneously, the emphasis young Chinese place on relationship depth drives them to maintain responsiveness toward important friends even amid fatigue, often prioritizing them in their interactions. Some also shift toward offline activities, using face-to-face gatherings or conversations to strengthen emotional bonds with close friends. Nineteen respondents felt that, due to social media fatigue, the quality of their friendships with close friends had remained stable or even deepened.

Now we basically eat lunch and dinner together almost every day, then go do activities together in the afternoon. We’re definitely closer than before, at least in a physical sense. We can also talk more—the amount of information conveyed through offline speech is faster than typing online, so conversations tend to be deeper (W2).

#### 5.2.2 Stagnation and fading of weak ties

In contrast to strong relationships, weak ties rely on more limited maintenance channels and often struggle to withstand the impact of burnout. For instance, common contemporary “like-based friendships” or “spark-based friendships” depend on high-frequency online interactions to sustain the connection. When one party becomes less enthusiastic about interacting or stops “keeping the spark alive,” the relationship loses its foundation for growth. Thirteen respondents indicated that when burnout leads to a decreased desire to socialize, the first group to be “abandoned” is that of weak ties. Thus, weak relationships tend to stagnate and fade during periods of fatigue.

I feel that people whose relationship quality is already well-established will not be greatly affected. At most, I might reduce socializing with people I wasn’t very close to begin with. But since we were not close to begin with, I would not feel regret or anything like that (W6).

## 6 Conclusion and discussion

Against the backdrop of the Internet’s deep integration into daily life, friendship among Chinese young people is increasingly influenced by mobile social media. On the one hand, social media facilitates the establishment and maintenance of friendships for this demographic. On the other, the pressure of maintaining a “permanent online presence” gradually brings negative effects. In particular, the emergence of social media fatigue has led to complex psychological and behavioral changes in the social interactions of this population. This study employs a mixed-methods approach combining quantitative and qualitative methods. First, based on a questionnaire survey, it reveals the patterns of association between social media burnout and friendship maintenance behaviors. Then, through in-depth interviews, it further explains the specific interaction strategies and behavioral choices individuals adopt in situations of burnout, in an attempt to answer a key question: What new effects does mobile social media use have on friendship quality among Chinese youth when they are experiencing a state of fatigue, and what are the underlying mechanisms of these effects/changes?

Findings reveal that changes in friendship quality among Chinese young people are not simply determined by the duration or frequency of use. Instead, they are closely linked to friendship maintenance behaviors moderated by social media fatigue. Specifically, fatigue can, to some extent, moderates young people’s willingness to engage and their response patterns in social interactions. This manifests as distancing from and filtering out weak ties, while concentrating limited energy on deeper relationships. Consequently, friendship networks exhibit a divergent pattern: “Strong ties tend to stabilize or deepen, while weak ties tend to stagnate or fade.”

From a traditional perspective, the breadth of friendship networks is often regarded as a key indicator for measuring an individual’s social support and mental health (Yao Yuan and Zhang Shun, 2016). However, this study challenges that assumption. Influenced by the instant-response culture of the social media era, some weak ties not only fail to provide valuable support and resources but can instead become sources of stress that trigger burnout due to factors like response obligations and social expectations. “More friends” no longer simply equates to “more resources”; it also means more messages, higher explanation costs, and greater psychological burdens. Consequently, when experiencing burnout, the youth of China first choose to reduce communication frequency and adopt passive responses to trim these loose, weak ties. In contrast, strong relationships—characterized by deep understanding and support—are prioritized for preservation and even strengthened during burnout periods. This shift reveals that in the social media era, young Chinese are increasingly evaluating friendship quality based on “depth” rather than the previous emphasis on “breadth.”

Although this study offers insights into the relationship between the intensity of mobile social media usage, social media fatigue, and friendship quality among young Chinese, several limitations exist. First, this study did not employ a fully validated multidimensional scale in the strict sense; some variables were measured using single-item items, and “social media burnout” was treated as a categorical variable in the statistical analysis. Consequently, the applicability of the questionnaire’s reliability and validity tests, as well as the results of the correlation analyses, are of limited persuasiveness. Second, the majority of respondents were Chinese college students sharing similar social environments, ages, and educational backgrounds, which somewhat limits the generalizability of the findings. Finally, the simplification of friendship quality into a three-category variable (“improved,” “deteriorated,” “remained largely unchanged”) inadequately accounts for variations in subdimensions such as emotional support, trust levels, and interaction satisfaction among the Chinese youth. To address these limitations, future research could adopt established social fatigue scales and multidimensional friendship quality measures to obtain more discriminative and granular data, thereby revealing the specific impacts of social fatigue on friendships. Additionally, expanding the sample size to include more diverse groups would enhance the generalizability of findings.

## Data Availability

The original contributions presented in the study are included in the article/supplementary material, further inquiries can be directed to the corresponding author.
